# S-nitrosylated PARIS Leads to the Sequestration of PGC-1α into Insoluble Deposits in Parkinson’s Disease Model

**DOI:** 10.3390/cells11223682

**Published:** 2022-11-19

**Authors:** Hanna Kim, Ji-Yeong Lee, Soo Jeong Park, Eunsang Kwag, Jihye Kim, Joo-Ho Shin

**Affiliations:** 1 Department of Pharmacology, Samsung Biomedical Research Institute, Sungkyunkwan University School of Medicine, Suwon 16419, Republic of Korea; 2 Single Cell Network Research Center, Sungkyunkwan University School of Medicine, Suwon 16419, Republic of Korea; 3 Samsung Biomedical Research Institute, Samsung Medical Center, Seoul 06351, Republic of Korea

**Keywords:** PARIS/ZNF746, S-nitrosylation, Parkinson’s disease, PGC-1α, nitrosative stress

## Abstract

Neuronal accumulation of parkin-interacting substrate (PARIS), a transcriptional repressor of peroxisome proliferator-activated receptor gamma coactivator 1-alpha (PGC-1α), has been observed in Parkinson’s disease (PD). Herein, we showed that PARIS can be S-nitrosylated at cysteine 265 (C265), and S-nitrosylated PARIS (SNO-PARIS) translocates to the insoluble fraction, leading to the sequestration of PGC-1α into insoluble deposits. The mislocalization of PGC-1α in the insoluble fraction was observed in S-nitrosocysteine-treated PARIS knockout (KO) cells overexpressing PARIS WT but not S-nitrosylation deficient C265S mutant, indicating that insolubility of PGC-1α is SNO-PARIS-dependent. In the sporadic PD model, α-synuclein preformed fibrils (α-syn PFFs)-injected mice, we found an increase in PARIS, SNO-PARIS, and insoluble sequestration of PGC-1α in substantia nigra (SN), resulting in the reduction of mitochondrial DNA copy number and ATP concentration that were restored by N(ω)-nitro-L-arginine methyl ester, a nitric oxide synthase (NOS) inhibitor. To assess the dopaminergic (DA) neuronal toxicity by SNO-PARIS, lentiviral PARIS WT, C265S, and S-nitrosylation mimic C265W was injected into the SN of either PBS- or α-syn PFFs-injected mice. PARIS WT and C265S caused DA neuronal death to a comparable extent, whereas C265W caused more severe DA neuronal loss in PBS-injected mice. Interestingly, there was synergistic DA loss in both lenti-PARIS WT and α-syn PFFs-injected mice, indicating that SNO-PARIS by α-syn PFFs contributes to the DA toxicity in vivo. Moreover, α-syn PFFs-mediated increment of PARIS, SNO-PARIS, DA toxicity, and behavioral deficits were completely nullified in neuronal NOS KO mice, suggesting that modulation of NO can be a therapeutic for α-syn PFFs-mediated neurodegeneration.

## 1. Introduction

Parkinson’s disease (PD) is the second most common neurodegenerative disease [1]. It has been estimated that PD increases with age, reaching a prevalence of 2.6% in people aged 85 to 89 years [1]. The causes of PD are largely divided into genetic and non-genetic (environmental factors) [2]. Various studies have been performed to understand the association between misfolded protein accumulation and PD development [3,4,5]. 

Previously, we showed that the Cys2His2 zinc finger protein ZNF746, also known as parkin-interacting substrate (PARIS), acts as a transcriptional repressor of peroxisome proliferator-activated receptor gamma coactivator 1-alpha (PGC-1α), a master regulator of mitochondrial biogenesis and function [6]. Inactivation of parkin leads to the accumulation of PARIS, resulting in the gradual loss of dopaminergic (DA) neurons in PD models [7]. Although quantitative changes in PARIS were reported in the substantia nigra (SN) of PD patients and several PD models, including conditional parkin knockout (KO) and α-synuclein preformed fibrils (α-syn PFFs)-injected mice [6,7,8,9], qualitative modifications of PARIS remain uncharacterized.

Nitric oxide (NO) is one of the gaseous signaling molecules involved in regulating various biological functions [10]. In normal conditions, NO production is tightly regulated, and high levels of NO due to increased neuronal NOS (nNOS) can trigger neuronal dysfunction by enhancing nitrosative stress, as observed in several neurodegenerative diseases, such as PD, Alzheimer’s disease (AD), Huntington’s disease (HD), and stroke [11]. The addition of NO to a cysteine residue within the target protein (S-nitrosylation) is a known mechanism that regulates protein structure and function [12]. S-nitrosylation of parkin leads to the inhibition of its ubiquitin E3 ligase activity [13]. Moreover, S-nitrosylation of the X-linked inhibitor of apoptosis (XIAP) leads to the impairment of its anti-caspase-3 activity and anti-apoptotic function [14]. S-nitrosylation of peroxiredoxin 2 (Prx2) inhibits its protective function, resulting in oxidative stress-induced neuronal cell death [15]. In addition, it has been reported that the activity of various transcription factors is regulated by S-nitrosylation, which promotes neuronal degeneration directly or indirectly [16]. 

Herein, we showed that PARIS is a novel substrate for S-nitrosylation, and S-nitrosylated PARIS (SNO-PARIS) translocates to the insoluble fraction along with PGC-1α, leading to mitochondrial dysfunction and DA neuronal cell death in a PD model.

## 2. Materials and Methods

### 2.1. Animal Experiments and Antibodies

All experiments were performed in a specific facility at Sungkyunkwan University School of Medicine (Suwon, Korea), and all procedures were approved by the Sungkyunkwan University School of Medicine Institutional Animal Care and Use Committee (IACUC, SKKUIACUC2021-07-36-1), following international guidelines. C57BL/6N mice were obtained from Orient (Suwon, South Korea) and maintained under a 12/12 h (hr) dark/light cycle in air-controlled rooms, with access to diet and water. All efforts were made to minimize animal suffering and reduce the number of animals used. Generation of PARIS KO mice was previously described [17], and neuronal NOS (nNOS) KO mice were purchased from the Jackson Laboratory (Bar Harbor, ME, USA: #002986). Detailed information about all antibodies used in this study is shown in the Supplementary Table S1.

### 2.2. Cell Culture and Transfection

Human neuroblastoma SH-SY5Y cells were grown in DMEM (Corning, Glendale, AZ, USA), supplemented with 10% fetal bovine serum (FBS, Corning, Glendale, AZ, USA) and antibiotics in a 5% CO_2_ atmosphere at 37 °C. SH-SY5Y cells were transfected using the X-tremeGENE DNA transfection reagent (Roche, Basel, Switzerland) in Opti-MEM medium (Life Technologies, Carlsbad, CA, USA), according to the manufacturer’s instructions. After 48 h, cells were lysed using the RIPA buffer (iNtRON Biotechnology, Seongnam, South Korea). 

For culturing primary mouse DA neurons, the protocol developed by Gaven et al. was used [18]. Briefly, E10 mouse embryos were collected, and the ventral mesencephalon was isolated from the embryonic brain. The isolated mesencephalon was transferred to a clear tube and washed with sterile PBS (1 mL) thrice. After the last wash, the isolated mesencephalon was incubated in Trypsin/EDTA (3 mL) for 15 min at 37 °C in a CO_2_ incubator. The Trypsin/EDTA solution was removed, followed by washing with the DMEM/F-12 Ham medium (Life Technologies, Carlsbad, CA, USA). The mesencephalon was homogenized using a fire-polished Pasteur pipette, and the homogenate was collected in a clear tube. Cells in the homogenate were counted and seeded onto poly-L-ornithine (PLO) coated 24-well plates with center plating (6 × 10^5^ cells/well). After 30 min (min), culture medium was added, and the plates were incubated in a 5% CO_2_ atmosphere at 37 °C. DA neurons were matured after culturing them 5–7 days in vitro (DIV). For transfection of primary mouse DA neurons, Lipofectamine 3000 (Invitrogen, Waltham, MA, USA) was used after 14–21 DIV.

### 2.3. Biotin Switch Technique

The biotin switch technique is modified from the study by Jaffery et al. [19]. Briefly, NO donor (GSNO or SNOC) treated cells were lysed in HEN buffer (250 mM HEPES-NaOH pH 7.7, 1 mM EDTA, 0.1 mM Neocuproine), and each sample was adjusted to the equal concentration. For blocking free cysteine residues, 20 mM MMTS (Sigma-Aldrich, St. Louis, MO, USA) in HEN buffer and 25% SDS (9:1 *v*/*v*) was added to the lysate and incubated for 20 min at 50 °C with frequent vortexing. Four volumes of cold acetone were added to each sample and incubated for 20 min at −20 °C to precipitate the proteins. Samples were collected by centrifugation at 3000× *g* for 10 min, and the pellet was gently washed with 70% acetone (4 × 1 mL) for 10 min. Following resuspension in the HENS buffer (HEN buffer with 1% SDS), 2.5 mg/mL biotin-HPDP (Thermo Fisher Scientific, Waltham, MA, USA), with 200 mM sodium ascorbate, were added, and the sample was rotated for 1 h at room temperature. Samples were precipitated with three volumes of cold acetone for 20 min at −20 °C and collected by centrifugation at 5000× *g* for 10 min. The pellet was gently washed with 70% acetone (4 × 1 mL) for 10 min. Following resuspension in 0.1 mL of HENS buffer per mg of protein in the initial protein sample and two volumes of neutralization buffer (20 mM HEPES-NaOH pH 7.7, 100 mM NaCl, 1 mM EDTA, 0.5% Triton X-100), samples were transferred to a fresh tube containing beads. The samples were rotated for 12–18 h at 4 °C. Beads were collected by centrifugation at 200× *g* and washed with the wash buffer (4 × 1 mL). The elution buffer (20 mM HEPES-NaOH pH 7.7, 100 mM NaCl, 1 mM EDTA, 100 mM β-mercaptoethanol) was added, and protein was eluted at room temperature, followed by centrifugation at 3000× *g* for 5 min. The supernatant was collected and mixed with the SDS-PAGE sample buffer.

### 2.4. Western Blot Analysis

SH-SY5Y cells were lysed with RIPA buffer (iNtRON Biotechnology, Seongnam, South Korea), and protein concentration was determined using the BSA protein assay kit (Thermo Fisher Scientific, Waltham, MA, USA). The protein samples were loaded onto either 8% or 10% SDS-PAGE gels. The separated proteins were transferred to nitrocellulose membranes (Bio-Rad, Hercules, CA, USA) and incubated in 5% skimmed milk for 30 min at room temperature. The membranes were incubated with primary antibodies overnight at 4 °C, followed by incubation with secondary antibodies for 1 h. After incubation with secondary antibodies, proteins were visualized using the enhanced chemiluminescence (ECL) substrate (Thermo Scientific, Waltham, MA, USA).

### 2.5. Real-Time Quantitative RT-PCR (qRT-PCR)

For measuring mRNA levels, total RNA was extracted using the QIAzol lysis reagent (Qiagen, Hilden, Germany). cDNA was synthesized using the Superscript III First-strand synthesis system (Invitrogen). qPCR mixture was prepared using the Rotor-Gene SYBR green PCR kit (Qiagen, Hilden, Germany), and qPCR was performed using Rotor-Gene Q (Qiagen, Hilden, Germany) according to the manufacturer’s instruction. The primer sequences are listed in the Supplementary Table S2.

### 2.6. mtDNA Copy Number Using qRT-PCR 

Total DNA was extracted with the QIAzol lysis reagent (Qiagen, Hilden, Germany). Relative quantities of mtDNA were analyzed using the Rotor-Gene Q real-time PCR (Qiagen, Hilden, Germany) and Rotor-Gene SYBR green PCR kit (Qiagen, Hilden, Germany), according to the manufacturer’s instructions. The primer sequences are listed in the Supplementary Table S2. 

### 2.7. ATP Measurement

The ENLITEN^®^ ATP Assay kit (Promega, Madison, WI, USA) was used for measuring ATP levels according to the manufacturer’s instructions. Briefly, cells were homogenized in 1% trichloroacetic acid (TCA) and centrifuged for 5 min at 18,000× *g* at 4 °C. The supernatant was subsequently neutralized by adding 2 M KOH (pH 7.75). The rLuciferase/Luciferin reagent was added to the sample, and luciferase activity was measured using a luminometer (GloMax, Promega, Madison, WI, USA).

### 2.8. Production of Lentivirus

For lentiviral production, 5 μg lentiviral Lenti-PGK vector harboring PARIS WT, PARIS C265S, PARIS C265W, 4 μg packaging vector Δ 8.9, and 1 μg VSVG envelope glycoprotein vector were co-transfected into HEK 293T cells using Fugene HD (Promega). Supernatants containing the lentivirus were harvested 36–48 h after transfection and ultracentrifuged at 25,000 rpm to concentrate the lentivirus. The pellet was resuspended in phosphate-buffered saline (PBS) and stored at −80 °C. Lentiviral transduction into SH-SY5Y cells was confirmed by puromycin (1 μg/mL) treatment.

### 2.9. Tissue Preparation for Histochemistry

For tissue preparation, mice were anesthetized with an intramuscular injection of a mixture of ketamine (100 mg/kg) and xylazine (10 mg/kg) and perfused with PBS, followed by perfusion with 4% paraformaldehyde in PBS. Mice brains were removed and post-fixed overnight at 4 °C. The brains were immersed in a 30% sucrose solution and stored at 4 °C until sectioning. Frozen brains were sectioned along the coronal plane (35 μm) using a microtome (HM430, Fisher Scientific, Waltham, MA, USA) and maintained in a storage solution at 4 °C.

### 2.10. Preparation of α-Synuclein Preformed Fibrils 

Recombinant mouse α-syn protein was purified as described by Polinski et al. [20]. The α-syn PFFs were prepared in PBS by constantly agitating 5 mg/mL α-syn monomer with a thermomixer (1000 rpm at 37 °C) (Eppendorf, Stevenage, UK) for 7 days. The α-syn aggregates were diluted to 2.5 mg/mL with PBS and sonicated for 30 sec (1 sec pulse on and off) at 20% amplitude (Branson Digital Sonifier 450, Fisher Scientific, Waltham, MA, USA) before use.

### 2.11. Immunofluorescence

Brain sections were fixed in 4% paraformaldehyde for 10 min and permeabilized with 0.1% Triton X-100 (*v*/*v*) in PBS for 10 min at room temperature, followed by incubation in blocking buffer (10% normal goat serum, 0.1% Triton X-100 in PBS) for 1 h. After three times of washing with PBS for 10 min, sections were incubated with primary antibodies overnight at 4 °C. The next day, sections were washed three times with PBS for 10 min and incubated with Alexa Fluor-conjugated secondary antibodies (Abcam, Cambridge, MA, USA) for 1 h at room temperature. Images were acquired using a fluorescence microscope (CTR6000, LEICA biosystems, Danvers, MA, USA).

### 2.12. Immunohistochemistry

Brain sections were blocked with the blocking solution (0.2% Triton X-100, 0.02% sodium azide, 10% normal goat serum in PBS) for 30 min at room temperature, followed by incubation with the rabbit polyclonal anti-tyrosine hydroxylase (TH; 1:1000 dilution) antibody overnight at 4 °C. The next day, sections were washed three times with PBS for 10 min and incubated with secondary antibodies for 1 h at room temperature. Sections were washed three times with PBS for 10 min and incubated in Vectastain Elite ABC reagent (Vector lab, Newark, CA, USA) for 30 min at room temperature. Each section was stained with diaminobenzidine (DAB kit, Vector lab), and images were acquired using a fluorescence microscope (CTR6000, LEICA biosystems, Danvers, MA, USA).

For Nissl staining, sections were mounted onto slides and stained with 0.5% cresyl violet (*w*/*v*), dehydrated through graded alcohols (70%, 80%, 90%, and 100% (vol/vol)), placed in xylene, and covered with a coverslip after the addition of DPX (Sigma Aldrich, St. Louis, MO, USA). To quantify the volume of each brain region, each section was analyzed using a fluorescence microscope (CTR6000, LEICA biosystems, Danvers, MA, USA). 

### 2.13. Purification of GST-PARIS Recombinant Protein

Full-length PARIS WT (aa 1-644) and XIAP were cloned into the pGEX-6p-1 vector that was then used to transform BL21 gold E. coli cells (Agilent Technologies, Santa Clara, CA, USA). The transformed cells were grown at 37 °C until the optical density reached 0.6, followed by cooling on ice for 1 h. The transformed E.coli cells were further grown in the presence of 0.5 mM isopropyl β-D-thiogalactopyranoside (IPTG) for 3 h at 37 °C. Cells were then harvested by centrifugation at 10,000× *g* for 5 min at 4 °C and lysed by sonication in the lysis buffer (4 M NaCl, 10% NP40, 1 M Tris-HCl pH 7.4, 0.5 M EDTA, 0.25 M EGTA, 0.1% β-mercaptoethanol, and 100 mM PMSF). The protein samples were centrifuged at 10,000 × *g* for 15 min, mixed with glutathione sepharose 4B (Sigma-Aldrich, St. Louis, MO, USA), and incubated at 4 °C overnight. The beads were then washed using the wash buffer (4 M NaCl, 10% NP40, 1 M Tris-HCl pH 7.4, 0.5 M EDTA, and 0.25 M EGTA) on ice, and the bound proteins were eluted with the elution buffer (40 mM glutathione, 1 M HEPES, 1 M NaCl, 100% glycerol, and 10% Triton X-100) for 1 h at 4 °C. The purity of GST-PARIS and GST-XIAP recombinant proteins were analyzed by SDS-PAGE, and concentration was determined using the Bradford assay.

### 2.14. Stereotaxic Injection

Stereotaxic injection was given, as previously described [6]. For α-syn PFFs injection, 8-week-old C57BL/6N mice (Orient, Suwon, South Korea) were anesthetized with a mixture of ketamine (100 mg/kg) and xylazine (10 mg/kg) and injection was given stereotaxically into the striatum with the following coordinates: anteroposterior (AP) = +0.2 mm, mediolateral (ML) = +2.0 mm, and dorsoventral (DV) = +2.8 mm from the bregma according to the mouse brain atlas. The injection volume used was 2 μL with an injection rate of 0.4 μL/min. For lentiviral vector injection, 8-week-old C57BL/6N mice were injected in the substantia nigra (SN) region with the following coordinates: anteroposterior (AP) = −3.0 mm, mediolateral (ML) = +1.2 mm, and dorsoventral (DV) = +4.3 mm from the bregma according to the mouse brain atlas. The injection volume used was 2 μL, with an injection rate of 0.4 μL/min.

### 2.15. Pole Test

The pole test was performed, as previously described, with minor modifications [21,22]. A metal rod (9 mm diameter, 50–60 cm long) was wrapped with a bandage gauze and placed inside the home cage. The mice were placed on the top of the pole (3 inches from the top of the pole) facing head-up. The total time taken to reach the base of the pole was measured. Before the actual test, the mice were trained for three consecutive days, and each training session consisted of three test trials. On the day of the test, mice were evaluated in three tests, and total time was recorded. The maximum cutoff to stop the test and recording was 120 s.

### 2.16. Rotarod Test

The rotarod test was performed, as previously described, with minor modifications [21,22]. The mice were placed on a rotating rod and trained for three consecutive days before the test. The speed of the rotating rod was gradually increased from 4 to 40 rpm over a 5 min period. The time taken by the mice to fall from the rod (latency) was recorded. The animals were tested in three trials per day for three consecutive days. The average latency of falling off the rod was recorded.

### 2.17. Statistical Analysis

Results are presented as mean ± standard error of the mean. Comparison between two groups was performed using the unpaired two-tailed Student’s *t*-test. The one-way ANOVA and two-way ANOVA, followed by Bonferroni’s post-test, were used for comparing three or more groups, respectively. A *p*-value < 0.05 was considered statistically significant.

## 3. Results

### 3.1. PARIS Is S-nitrosylated at Cysteine 265 Residue

**Figure 1:**
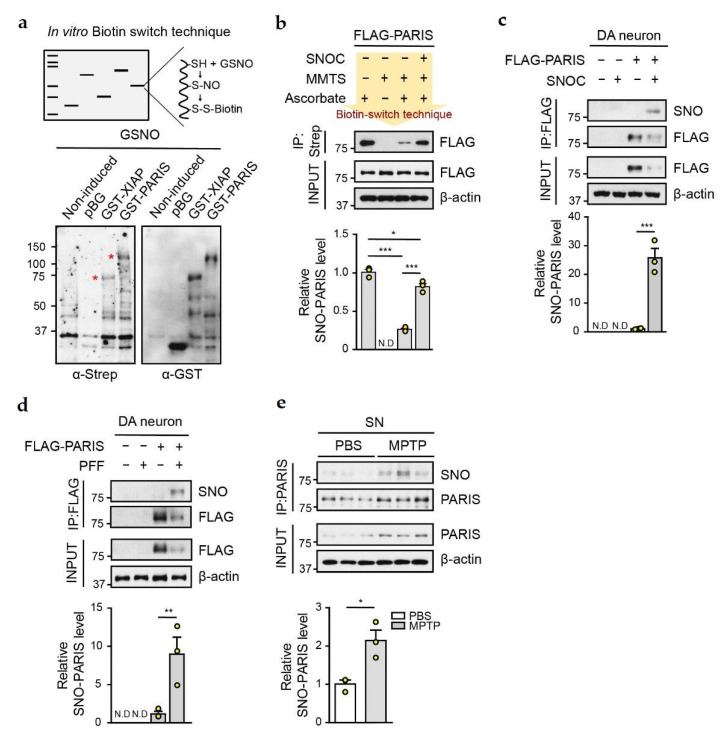
PARIS can be S-nitrosylated. (**a**) S-nitrosylation of PARIS using the in vitro biotin switch technique. Illustration depicting the process of biotin switch at the S-nitrosylation site (upper panel). Recombinant GST protein, GST-XIAP (S-nitrosylation positive control), and GST-PARIS were exposed to the NO donor and GSNO (200 μM) for 30 min, followed by biotin switch technique. The S-nitrosylated recombinant protein was evaluated using streptavidin. Red asterisks indicate S-nitrosylated proteins (bottom panel). (**b**) In vitro biotin switch technique using FLAG-tagged PARIS. The lysate of SH-SY5Y cells overexpressing FLAG-PARIS was incubated for 30 min with or without SNOC (50 μM), and immunoprecipitation (IP) was performed using streptavidin. SNO-PARIS level was evaluated using the anti-FLAG antibody. Negative control did not contain ascorbate. Bottom panel, relative levels of SNO-PARIS normalized to the FLAG input, *n* = 3, one-way ANOVA followed by Tukey’s multiple comparison test, ** *p* < 0.01, *** *p* < 0.001, N.D, not detected. (**c**) S-nitrosylation of PARIS in primary DA neurons. Cell lysates from primary DA neurons expressing FLAG-PARIS were incubated with SNOC (50 μM), and immunoprecipitation was performed with the anti-FLAG antibody. SNO-PARIS was detected using the anti-SNO antibody. Bottom panel, relative levels of SNO-PARIS normalized to immunoprecipitated FLAG-PARIS levels, *n* = 3, one-way ANOVA followed by Tukey’s multiple comparison test, *** *p* < 0.001, N.D, not detected. (**d**) Increase in SNO-PARIS levels by α-syn PFFs treatment in DA neurons. Primary DA neurons expressing FLAG-PARIS were exposed to α-syn PFFs (0.1 mg/mL α-syn PFFs diluted in media with a ratio of 1:25) for 2 weeks. Cells were then lysed, and immunoprecipitation was performed with the anti-FLAG antibody. SNO-PARIS was detected using the anti-SNO antibody. Bottom panel, relative SNO-PARIS levels normalized to immunoprecipitated FLAG-PARIS levels, *n* = 3, one-way ANOVA followed by Tukey’s multiple comparison test, ** *p* < 0.01, N.D, Not detected. (**e**) Representative immunoblot showing increased levels of SNO-PARIS in the SN region of MPTP-induced mice. SN lysates were prepared, and immunoprecipitation was performed using the anti-PARIS antibody. SNO-PARIS was detected using the anti-SNO antibody. Bottom panel, relative SNO-PARIS levels normalized to immunoprecipitated PARIS levels, *n* = 3, Student’s *t*-test, * *p* < 0.05.

To investigate whether PARIS could be S-nitrosylated in vitro, we generated GST-tagged PARIS and XIAP (positive control) recombinant proteins. Membrane-bound GST, GST-XIAP, and GST-PARIS proteins were exposed to 200 μM S-nitrosoglutathione (GSNO, NO donor), following which the biotin switch technique was applied (Figure 1a). This technique enabled us to detect biotin-conjugated PARIS and XIAP, but not GST alone, indicating that PARIS can be S-nitrosylated in vitro (Figure 1a). To evaluate S-nitrosylation of PARIS, SH-SY5Y cells overexpressing Flag-tagged PARIS were exposed to 50 μM S-nitrosocysteine (SNOC, NO donor) for 30 min, following which cells were lysed, and lysates were subjected to biotin switch technique (Figure 1b). Briefly, the lysates were blocked with methyl methanethiosulfonate (MMTS, blocking reagent) and incubated with ascorbate for biotin conjugation. S-nitrosylated proteins were precipitated using streptavidin-bound agarose beads, and S-nitrosylated Flag-PARIS (SNO-PARIS) was detected using the HRP-conjugated anti-Flag antibody. Results demonstrated that Flag-tagged PARIS was slightly S-nitrosylated, and its level was increased by SNOC treatment (Figure 1b). 

Next, we tested whether S-nitrosylation of PARIS can occur in DA neurons. Primary mouse DA neurons transfected with Flag-tagged PARIS were exposed to SNOC (50 μM) for 30 min. Cells were then lysed, immunoprecipitation was performed with the anti-Flag antibody, and immunoblot analysis was performed with the anti-S-nitrosocysteine (anti-SNO) antibody to evaluate the levels of SNO-PARIS. We observed that the level of SNO-PARIS was significantly increased upon SNOC treatment (Figure 1c). Similar results were observed in α-syn PFFs-treated DA neurons (Figure 1d), suggesting that α-syn PFFs and NO donor trigger S-nitrosylation of PARIS in DA neurons.

Since the level of SNO-PARIS was increased in the presence of NO donor and α-syn PFFs, we further evaluated the levels of SNO-PARIS in the SN of MPTP-administered mice, a well-characterized PD model. Total PARIS was immunoprecipitated using the anti-PARIS antibody and subjected to immunoblot analysis with the anti-SNO antibody, showing that MPTP administration led to a greater than two-fold increase in PARIS and SNO-PARIS in the SN compared to PBS administration (Figure 1e). 

**Figure 2:**
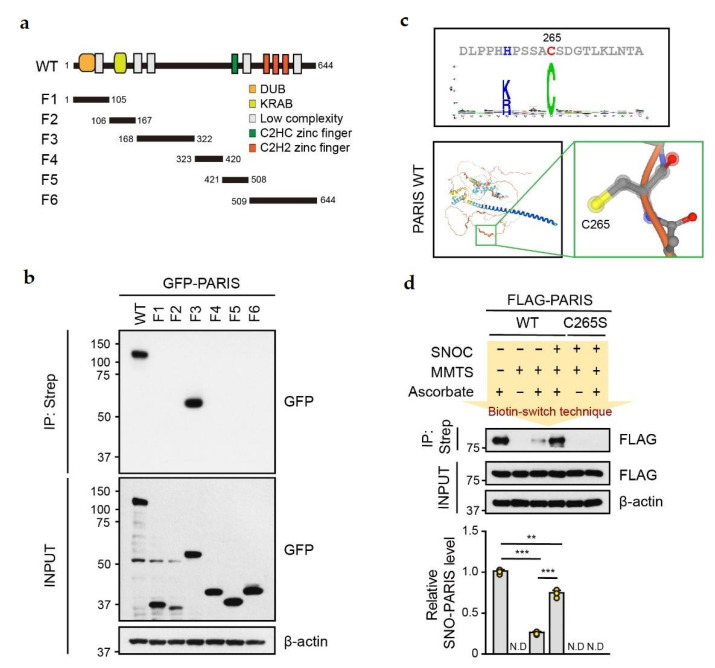
The cysteine 265 residue is an important site for S-nitrosylation of PARIS. (**a**) Schematic diagram of PARIS WT and truncated fragments of PARIS. F1, 1–105; F2, 106–167; F3, 168–322; F4, 323–420; F5, 421–508; F6, and 509–644. (**b**) Representative immunoblots showing S-nitrosylation of PARIS WT and F3 truncated fragment. Lysates from SH-SY5Y cells expressing GFP-tagged PARIS WT and fragments (F1-F6) were exposed to SNOC (50 μM) for 30 min and subject to immunoprecipitation using streptavidin. SNO-PARIS was detected using the anti-GFP antibody. (**c**) Computational prediction suggesting that cysteine 265 residue is the S-nitrosylation site on PARIS. (**d**) Lysates from SH-SY5Y cells expressing FLAG-PARIS WT and S-nitrosylation deficient mutant C265S were incubated with or without SNOC (50 μM) for 30 min, and SNO-PARIS was detected using the biotin switch technique (*n* = 3) and one-way ANOVA was followed by Tukey’s multiple comparison test, ** *p* < 0.01, *** *p* < 0.001, N.D, not detected.

To identify the S-nitrosylation site in PARIS, six GFP-tagged PARIS truncated fragments (F1~F6) were generated (Figure 2a) and transfected into SH-SY5Y cells. Cells were then lysed, and lysates were treated with SNOC (50 μM, 30 min) for in vitro S-nitrosylation (Figure 2b). Strong signals of S-nitrosylation were observed in lysates from GFP-tagged PARIS WT and F3 expressing cells (Figure 2b). Next, we predicted the possible S-nitrosylation site in PARIS using the GPS-SNO program (http://sno.biocuckoo.org/ (accessed on 18 August 2018)) [23], revealing that cysteine 265 (C265) within the PARIS F3 fragment is a putative target residue for S-nitrosylation (Figure 2c, upper panel). Three-dimensional structuring by bioinformatic analysis (https://alphafold.ebi.ac.uk/ (accessed on 15 November 2022)) predicted that C265 residue is exposed (Figure 2c, bottom panel) [24], demonstrating that C265 within the PARIS F3 fragment is a putative S-nitrosylation site. To verify this, the Flag-tagged PARIS S-nitrosylation-deficient mutant, C265S, was transfected into SH-SY5Y cells. Cells were lysed followed by treatment with SNOC (50 μM, 30 min) for biotin switch. Notably, S-nitrosylation of PARIS WT was robust, whereas PARIS C265S failed to be S-nitrosylated under SNOC-treated conditions (Figure 2d). Taken together, our results suggest that C265 residue is the S-nitrosylation site in PARIS.

### 3.2. SNO-PARIS Translocates to the Insoluble Fraction

**Figure 3:**
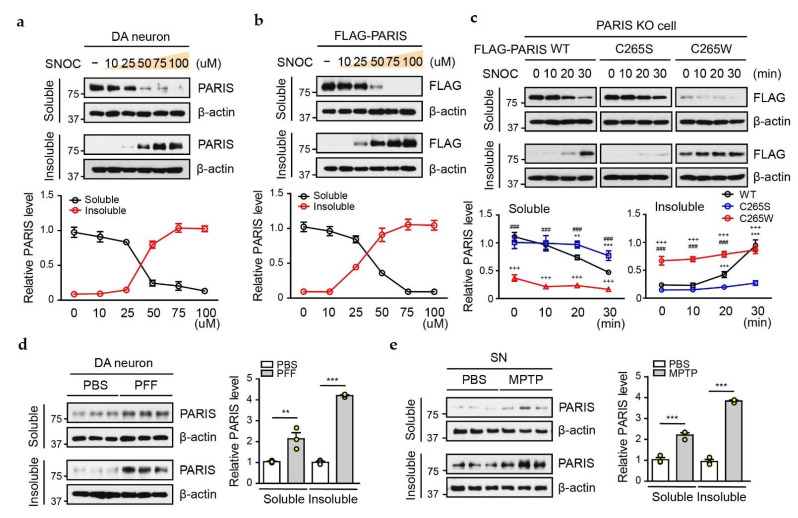
S-nitrosylated PARIS translocates to the insoluble fraction. (**a**) PARIS translocation to the insoluble fraction by SNOC. Cell lysates from primary DA neurons were exposed to different concentrations of SNOC (0–100 μM) for 30 min and then separated into RIPA-soluble and RIPA-insoluble fractions. Bottom panel, relative PARIS levels normalized to β-actin, *n* = 3. (**b**) Lysates from SH-SY5Y cells expressing FLAG-tagged PARIS were exposed to different concentrations of SNOC (0–100 μM) for 30 min and then separated into RIPA soluble and RIPA insoluble fractions. Bottom panel, relative PARIS levels normalized to β-actin, *n* = 3. (**c**) Representative immunoblots showing time-dependent alteration in PARIS solubility. SH-SY5Y PARIS KO cells expressing FLAG-PARIS WT, S-nitrosylation deficient mutant (C265S) and mimic mutant (C265W) were exposed to SNOC (50 μM) for the indicated time (0–30 min), and their lysates were then divided into RIPA-soluble and RIPA-insoluble fractions. PARIS levels in the soluble (left bottom panel) and insoluble fractions (right bottom panel) normalized to β-actin (*n* = 3). Two-way ANOVA was followed by Bonferroni’s post-test, WT vs. C265S; ** *p* < 0.01, *** *p* < 0.001, WT vs. C265W; ### *p* < 0.001, C265S vs. C265W; +++ *p* < 0.001. (**d**) Cell lysates from primary DA neurons were treated with α-syn PFFs (0.1 mg/mL α-syn PFFs diluted in media with a ratio of 1:25) for 2 weeks and then separated into RIPA-soluble and RIPA-insoluble fractions. PARIS was detected using the anti-PARIS antibody. Right panel—relative PARIS levels normalized to β-actin, *n* = 3, one-way ANOVA followed by Tukey’s multiple comparison test, ** *p* < 0.01, *** *p* < 0.001. (**e**) Lysates from the SN region of PBS- and MPTP-administered mice were separated into RIPA soluble and RIPA insoluble fractions, followed by immunoblot assay with anti-PARIS antibody. Right panel—relative PARIS level normalized to β-actin, *n* = 3. One-way ANOVA followed by Tukey’s multiple comparison test, *** *p* < 0.001.

To understand the pathophysiological role of SNO-PARIS, we investigated PARIS distribution in the RIPA-soluble and insoluble fractions of DA neurons treated with SNOC at different concentrations (0–100 µM) for 30 min (Figure 3a). PARIS level was decreased gradually in the soluble fraction, whereas it was increased in the insoluble fraction in a dose-dependent manner (Figure 3a). Similar results were observed in SNOC-treated SH-SY5Y cells overexpressing Flag-tagged PARIS (Figure 3b). To confirm whether the translocation of PARIS to the insoluble fraction is S-nitrosylation-dependent, we transfected Flag-tagged PARIS WT, C265S, and the S-nitrosylation-mimic mutant (C265W) into PARIS KO SH-SY5Y cells and treated them with SNOC (50 µM) for the indicated time. Unlike PARIS WT, the distribution of PARIS C265S was low, whereas that of PARIS C265W was high in the insoluble fraction without SNOC treatment (Figure 3c). Minute levels of PARIS C265S in the insoluble fraction and C265W in the soluble fraction might be due to off-target S-nitrosylation on another cysteine residue or due to saturation, respectively. We also observed an increase in PARIS in the insoluble fraction of α-syn PFFs-treated DA neurons (Figure 3d) and SN of MPTP-administered mice (Figure 3e), suggesting that the transition of SNO-PARIS to the insoluble fraction could be an important mechanism underlying PARIS-mediated toxicity. 

### 3.3. SNO-PARIS Sequesters PGC-1α in the Insoluble Fraction

**Figure 4:**
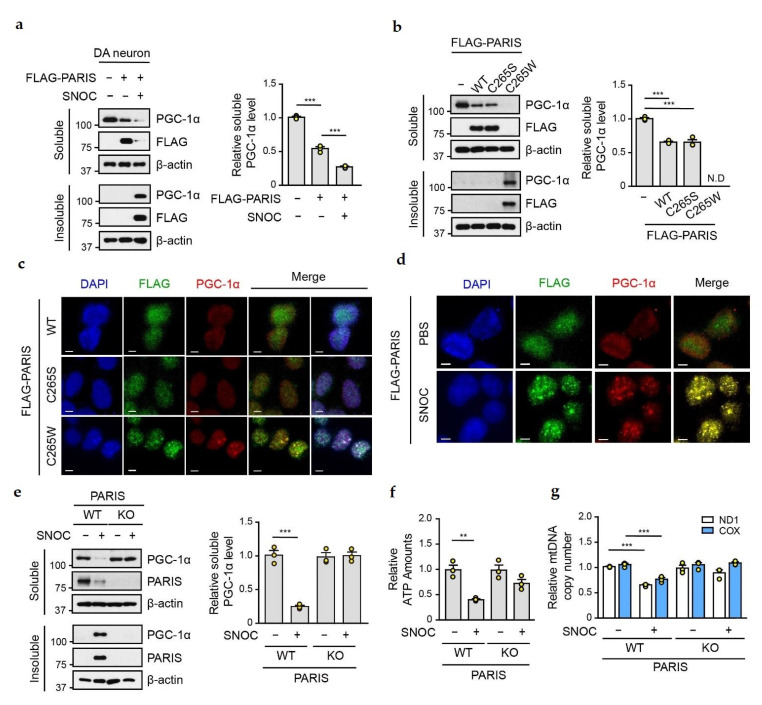
SNO-PARIS sequesters PGC-1α in the insoluble fraction. (**a**) Cell lysates from primary DA neurons expressing FLAG-tagged PARIS were incubated with or without SNOC (50 μM) for 30 min and then separated into RIPA-soluble and RIPA-insoluble fractions, followed by immunoblot assay. Right panel, relative PGC-1α level in the soluble fraction normalized to β-actin (*n* = 3). One-way ANOVA followed by Tukey’s multiple comparison test, *** *p* < 0.001. (**b**) Lysates of SH-SY5Y cells overexpressing FLAG-tagged PARIS WT and mutants (C265S, C265W) were separated into RIPA-soluble and RIPA-insoluble fractions, followed by immunoblot assay. Right panel, relative PGC-1α level in the soluble fraction normalized to β-actin (*n* = 3). One-way ANOVA followed by Tukey’s multiple comparison test, *** *p* < 0.001. N.D, not detected. (**c**) Representative immunofluorescence images of the FLAG-tagged PARIS WT and mutants (C265S, C265W) expressing SH-SY5Y cells. PARIS in green and endogenous PGC-1α in red. Scale bar = 5 μm. (**d**) Representative immunofluorescence images of SNOC (50 μM, 30 min) treated SH-SY5Y cells showing FLAG-tagged PARIS WT (green) and endogenous PGC-1α (red) expression. Scale bar = 5 μm. (**e**) Lysates of PARIS Knockout (KO) SH-SY5Y cells treated with SNOC (50 μM) for 30 min were separated into RIPA-soluble and RIPA-insoluble fractions, followed by immunoblot assay. Endogenous PARIS and PGC-1α levels were detected with indicated antibodies. Right panel, relative PGC-1α level in the soluble fraction normalized to β-actin level (*n* = 3). Two-way ANOVA followed by Bonferroni’s post-test, *** *p* < 0.001. (**f**) Measurement of relative ATP levels in PARIS KO cells treated with or without SNOC (50 μM) for 30 min using the ENLITEN luciferase assay (*n* = 3). Two-way ANOVA followed by Bonferroni’s post-test, ** *p* < 0.01. (**g**) Relative mitochondrial DNA (mtDNA) copy number in PARIS KO cells treated with or without SNOC (50 μM) for 30 min. NADH dehydrogenase subunit 1 (ND1) and cytochrome C oxidase (COX) levels were normalized to β-actin level, *n* = 3 per group. Two-way ANOVA followed by Bonferroni post-test, *** *p* < 0.001.

Next, we evaluated the protein level of PGC-1α in primary mouse DA neurons transfected with Flag-tagged PARIS, following SNOC treatment (50 µM for 30 min). The expression of PGC-1α was reduced in the soluble fraction of PARIS overexpressing cells, and further reduction was observed upon SNOC treatment (Figure 4a). Since PGC-1α was detected in the insoluble fraction of SNOC-treated DA neurons (Figure 4a), we hypothesized that SNO-PARIS sequesters PGC-1α into the insoluble fraction under nitrosative stress conditions. To address this, we examined the levels of PGC-1α in the insoluble pellet of SH-SY5Y cells transfected with Flag-tagged PARIS WT, C265S, and C265W. As previously reported [6], we found that the overexpression of PARIS WT and C265S reduced the level of soluble PGC-1α, whereas S-nitrosylation-mimic C265W caused the translocation of the endogenous PGC-1α to the insoluble fraction (Figure 4b). In addition, immunofluorescence analysis showed that PARIS C265W was mainly distributed in the nuclear puncta with a strong signal of PGC-1α (Figure 4c). We also confirmed the localization of Flag-tagged PARIS and endogenous PGC-1α in the nuclear puncta of SNOC-treated SH-SY5Y cells (Figure 4d). Notably, immunoblot analysis confirmed the insolubility of PGC-1α in SNOC-treated PARIS WT cells, whereas sequestration of PGC-1α into the insoluble fraction was completely blocked in SNOC-treated PARIS KO cells (Figure 4e), indicating that insoluble confinement of PGC-1α is SNO-PARIS-dependent. 

To investigate the physiological readouts by the reduction of soluble PGC-1α under nitrosative stress conditions, we measured ATP levels and mitochondrial DNA copy numbers in SNOC-treated PARIS WT and KO cells. ATP levels were decreased (Figure 4f), and the levels of two different mitochondrial markers, NADH dehydrogenase subunit 1 (ND1) and cytochrome C oxidase (COX), were also reduced in SNOC-treated WT cells (Figure 4g). In contrast, the levels of ATP and mitochondrial biogenesis were unchanged by SNOC treatment in PARIS KO cells, suggesting that mitochondrial abnormalities by SNOC are dependent on the transition of SNO-PARIS and sequestration of PGC-1α into the insoluble fraction.

### 3.4. L-NAME Reduces the Levels of Insoluble SNO-PARIS and PGC-1α, and Protects the Dopaminergic Neurons from α-syn PFFs-Induced Toxicity

**Figure 5:**
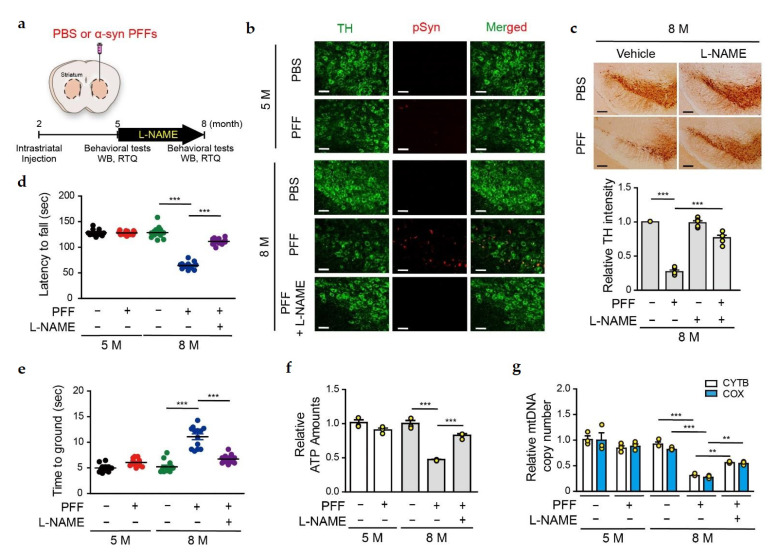
Nitrosative stress is responsible for DA neuronal cell death and motor dysfunction in α-syn PFFs injected sporadic PD mouse model. (**a**) Study design for intrastriatal PBS or α-syn PFFs injection. α-syn PFFs (5 μg/mouse) were stereotaxically injected into the striatum of C57BL/6 mice at the indicated time. L-NAME administration and behavioral testing were performed, as indicated in the timeline. All mice were sacrificed after the final behavioral experiments. (**b**) Representative immunofluorescence images showing TH (green) and phospho-α-syn (red) expression in the SN region of α-syn PFFs injected mice. Scale bar = 50 μm. (**c**) Representative images showing TH staining of the midbrain sections from the SN region of PBS- or α-syn PFFs-injected mice, with or without L-NAME treatment (6 months post-injection). Scale bar = 200 μm. Bottom panel, relative TH intensity in all mice groups evaluated using the imageJ software. *n* = 5, One-way ANOVA followed by Tukey’s multiple comparison test, *** *p* < 0.001. (**d**) Pole test of PBS- or α-syn PFFs-injected mice treated with L-NAME at the age of 5 and 8 months. *n* = 13 per group. Two-way ANOVA followed by Bonferroni’s post-test, *** *p* < 0.001. (**e**) Rotarod test of PBS- or α-syn PFFs-injected mice at the age of 5 and 8 months. *n* = 13 per group. Two-way ANOVA followed by Bonferroni’s post-test, *** *p* < 0.001. (**f**) Relative ATP levels in the SN region of 5- and 8-month old α-syn PFFs-injected mice evaluated using the ENLITEN luciferase assay. *n* = 3. Two-way ANOVA followed by Bonferroni’s post-test, *** *p* < 0.001. (**g**) Relative mitochondrial DNA (mtDNA) copy number in the SN region of 5- and 8-month old α-syn PFFs-injected mice. Relative levels of cytochrome b (CYTB) and cytochrome C oxidase (COX) normalized to GAPDH levels. *n* = 3. Two-way ANOVA followed by Bonferroni’s post-test, ** *p* < 0.01, *** *p* < 0.001.

Since the administration of α-syn PFFs increased the levels of soluble and insoluble PARIS in DA neurons (Figure 4a), we checked whether PARIS nitrosylation plays a deleterious role in α-syn PFFs-mediated DA neurons degeneration in vivo. α-syn PFFs were intrastriatally injected into 2-month-old C57BL/6 mice, and N(ω)-nitro-L-arginine methyl ester (L-NAME, NOS inhibitor) was given by drinking water (approximately 50 mg/kg/day) for 3 months prior to sacrifice, as illustrated (Figure 5a). To assess the α-syn PFFs-mediated toxicity, brain sections from 5- and 8-month-old α-syn PFFs-injected mice (at post-injection 3 and 6 months, respectively) were co-immunostained with tyrosine hydroxylase (TH) and phospho-α-syn-129 antibodies. Phosphorylated α-syn was minimally detected in the SN of 5-month-old α-syn PFFs-injected mice, whereas its signal was greater in the SN of 8-month-old α-syn PFFs-injected mice (Figure 5b), indicating that there was an age-dependent aggregation of α-syn PFFs as reported [25]. Notably, L-NAME administration significantly reduced the phospho-α-syn-129 signal in the SN of 8-month-old α-syn PFFs-injected mice (Figure 5b). Accordingly, stereological TH staining revealed a significant loss of DA neurons in the SN of 8-month-old, but not 5-month-old α-syn PFFs-injected mice (Figure 5c). Moreover, α-syn PFFs-mediated DA neuronal loss was prevented by L-NAME administration, implicating that nitrosative stress is involved in α-syn PFFs-mediated DA degeneration (Figure 5c). 

Next, the α-syn PFFs-injected mice were subjected to behavioral tests and then sacrificed for biochemical analysis (Figure 5a). We observed that the abnormal locomotor behavior in 8-month-old α-syn PFFs-injected mice, and this retardation in motor functions of the 8-month-old α-syn PFFs-injected mice, was significantly ameliorated by L-NAME administration (Figure 5d,e). Next, we measured the ATP concentration and mitochondrial DNA copy number in the SN of 5- and 8-month-old α-syn PFFs-injected mice. Results showed a reduction in ATP concentration and mitochondrial DNA copy number in the SN of 8-month-old α-syn PFFs-injected mice, and these changes in the 8-month-old mice were ameliorated by L-NAME administration (Figure 5f,g). These results suggest that NO exerts a deleterious effect in the α-syn PFFs-induced PD model, and modulating the NO signaling might be useful for preventing DA neurons degeneration.

### 3.5. PGC-1α Sequestration by α-syn PFFs Is SNO-PARIS-Mediated In Vivo

**Figure 6:**
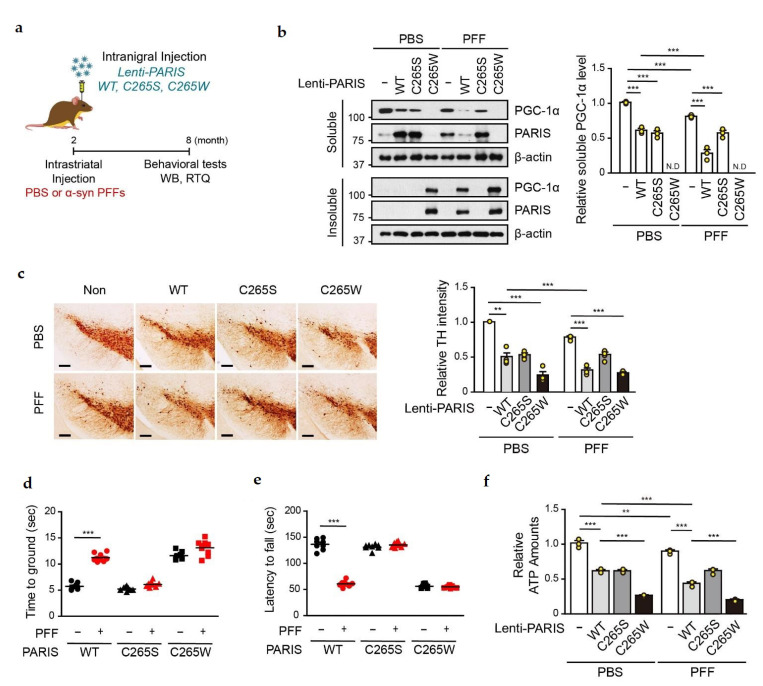
Loss of functional PGC-1α due to α-syn PFFs is SNO-PARIS-dependent. (**a**) Study timeline for intrastriatal α-syn PFFs injection and intranigral injection of lentiviruses (pLenti-GFP, pLenti-PARIS WT, pLenti-PARIS C265S, and pLenti-PARIS C265W). Biochemical and behavioral testing was performed as indicated in the timeline, and all mice were sacrificed after behavioral experiments. (**b**) PARIS and PGC-1α levels in RIPA-soluble and RIPA-insoluble fractions of the SN region of α-syn PFFs/lentiviruses injected mice. Right panel—relative PGC-1α level in the soluble fraction normalized to β-actin level. *n* = 3. Two-way ANOVA followed by Bonferroni’s post-test, *** *p* < 0.001. N.D, not detected. (**c**) Representative TH staining of the midbrain sections from the SN region of α-syn PFFs/lentiviruses-injected mice. Scale bar = 200 μm. Right panel—relative TH intensity was measured by using the ImageJ software. *n* = 4. Two-way ANOVA, followed by Bonferroni’s post-test, ** *p* < 0.01, *** *p* < 0.001. Pole test (**d**) and rotarod test (**e**) of α-syn PFFs/lentiviruses-injected mice at the age of 8 months. *n* = 8–9 per group. Two-way ANOVA followed by Bonferroni’s post-test, *** *p* < 0.001. (**f**) Relative ATP levels in the SN region of α-syn PFFs/lentiviruses-injected mice using the ENLITEN luciferase assay. *n* = 3. Two-way ANOVA followed by Bonferroni’s post-test, ** *p* < 0.01, *** *p* < 0.001.

To investigate whether SNO-PARIS plays a role in the α-syn PFFs-induced PD model, we stereotaxically injected both α-syn PFFs and lentiviruses overexpressing PARIS WT, C265S, or C265W into the STR and SN of 2-months-old C57BL/6 mice, respectively (Figure 6a). After 6 months of lentiviral injection, mice were subjected to behavioral tests and then sacrificed for further analysis. We investigated the levels of PARIS and PGC-1α in the insoluble fraction of SN from mice co-injected with α-syn PFFs and PARIS lentiviruses to determine whether PGC-1α sequestration by α-syn PFFs is SNO-PARIS-mediated in vivo (Figure 6b). Both PARIS C265W and PGC-1α were shifted into the insoluble fraction in the SN of PBS-injected mice, and the administration of α-syn PFFs increased the levels of PARIS WT and PGC-1α in the insoluble compartment but not PARIS C265S (Figure 6b), indicating that S-nitrosylation of PARIS is required for the transition of PGC-1α to the insoluble fraction in vivo. 

Since the level of functional PGC-1α was significantly reduced by SNO-PARIS, we monitored the loss of DA neurons by TH staining in the SN of α-syn PFFs-injected mice overexpressing PARIS WT and mutants. Results showed a significantly higher DA neuronal death in the SN of α-syn PFFs-administered mice overexpressing PARIS WT but not C265S compared to PBS-administered mice overexpressing PARIS WT (Figure 6c). Moreover, mice overexpressing PARIS C265W showed severe DA neuronal loss even in the absence of α-syn PFFs, and the cell loss was comparable to that observed in α-syn PFFs-administered mice overexpressing PARIS WT (Figure 6c). These results suggest that PARIS nitrosylation exerts the toxic effects of α-syn PFFs on DA neurons (Figure 6c). 

Next, we performed the pole test and rotarod test to examine whether S-nitrosylation of PARIS at cysteine 265 is associated with motor dysfunctions observed in α-syn PFFs-administered mice. Results showed that there were no additional behavioral abnormalities in α-syn PFFs administered mice overexpressing PARIS C265S compared to PBS-administered mice with PARIS C265S overexpression (Figure 6d,e). In contrast, mice overexpressing PARIS WT were more vulnerable to α-syn PFFs toxicity and showed abnormal motor functions similar to those observed in C265W overexpressing mice (Figure 6d,e). Moreover, we confirmed mitochondrial dysfunction by measuring the ATP concentration in the SN of PARIS WT, C265S, and C265W overexpressing mice injected with α-syn PFFs (Figure 6f). These results suggest that S-nitrosylation of PARIS at cysteine 265 contributes to mitochondrial deficits and motor dysfunction in α-syn PFFs-injected PD model mice. 

### 3.6. Amelioration of α-syn PFFs-Medicated Toxicity in the SN of nNOS KO Mice

**Figure 7:**
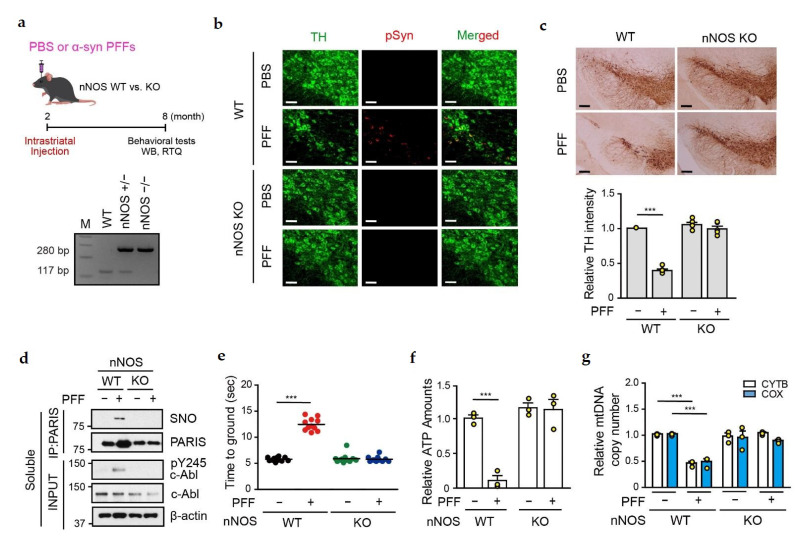
α-syn PFFs toxicity to DA neurons were completely nullified in nNOS KO mice. (**a**) Study timeline for intrastriatal PBS or α-syn PFFs injection in nNOS KO mice. The α-syn PFFs (5 μg) were administered in both WT and nNOS KO mice for 6 months. Behavioral tests were performed as indicated in the timeline, and all mice were sacrificed after the behavioral experiments. (**b**) Representative immunofluorescence images showing TH (green) and anti-phospho-α-syn (red) expression in the SN region of PBS- or α-syn PFFs-injected WT and nNOS KO mice. Scale bar = 50 μm. (**c**) Representative TH staining of the midbrain sections of α-syn PFFs-injected WT and nNOS KO mice. Scale bar = 200 μm. Bottom panel, relative TH intensity in different mice groups evaluated using the ImageJ software. *n* = 5. Two-way ANOVA followed by Bonferroni’s post-test, *** *p* < 0.001. (**d**) SN lysates of α-syn PFFs-injected WT and nNOS KO mice were subject to immunoprecipitation and immunoblot assay with the indicated antibodies. (**e**) Pole test of α-syn PFFs-injected WT and nNOS KO mice. *n* = 11 per group. Two-way ANOVA followed by Bonferroni’s post-test, *** *p* < 0.001. (**f**) Relative ATP levels in the SN region of PBS- or α-syn PFFs-injected WT and nNOS KO mice evaluated using the ENLITEN luciferase assay. *n* = 3. Two-way ANOVA followed by Bonferroni’s post-test, *** *p* < 0.001. (**g**) Relative mitochondrial DNA (mtDNA) copy number in the SN region PBS- or α-syn PFFs-injected WT and nNOS KO mice. Relative levels of cytochrome b (CYTB) and cytochrome C oxidase (COX) normalized to GAPDH levels. *n* = 3. Two-way ANOVA followed by Bonferroni’s post-test, *** *p* < 0.001.

Since the NOS blocker, L-NAME, prevented the α-syn PFFs-mediated DA neuronal cell death (Figure 5), we stereotaxically injected α-syn PFFs into the striatum of neuronal NOS knockout (nNOS KO) mice to assess whether nitrosative stress is essential for α-syn PFFs-mediated neurotoxicity (Figure 7a). nNOS KO was confirmed by genotyping by PCR technique (Figure 7a). Co-immunostaining with TH and phospho-α-syn-129 antibodies was performed to monitor α-syn aggregation in the SN of PBS- and α-syn PFFs-administered nNOS KO mice at 6 months post-injection. Phospho-α-syn-129 signal was successfully detected in TH-positive cells of α-syn PFFs-administered nNOS WT mice, but not in α-syn PFFs-administered nNOS KO mice (Figure 7b). Moreover, no DA neuronal cell death was observed in the SN of α-syn PFFs-injected nNOS KO mice (Figure 7c), indicating that nitrosative stress is an important factor for α-syn PFFs-mediated neurotoxicity. 

Interestingly, SNO-PARIS was undetectable in the SN of α-syn PFFs-administered nNOS KO mice (Figure 7d). Since PARIS accumulation by α-syn PFFs was observed in the SN of α-syn PFFs-administered WT, but not nNOS KO mice, we evaluated the level of phosphorylated c-Abl (Y245), an indicator of c-Abl activation, which inhibits parkin leading to PARIS accumulation [26]. Phosphorylated c-Abl was detected in the SN of α-syn PFFs-administered WT, but not in nNOS KO mice, explaining no accumulation of PARIS in the nNOS KO mice (Figure 7d). These results suggest that nitrosative stress activates c-Abl leading to parkin inhibition, PARIS accumulation, and S-nitrosylation of PARIS. Moreover, the administration of α-syn PFFs failed to cause behavioral deficits and mitochondrial abnormalities in the SN of nNOS KO mice (Figure 7e–g). 

## 4. Discussion

Herein, we showed that S-nitrosylation of PARIS at cysteine 265 regulates the localization of PARIS and solubility of PGC-1α and contributes to DA neuronal cell death. Although the cysteine 265 residue of PARIS is outside the protein functional domains, such as KRAB and zinc finger domains, S-nitrosylation at this residue is responsible for the translocation of PARIS to the insoluble fraction. Furthermore, SNO-PARIS interacts with PGC-1α under nitrosative stress conditions, leading to the sequestration of soluble PGC-1α into the insoluble fraction. We also demonstrated that α-syn PFFs-induced DA neuronal cell death, it can be rescued by L-NAME administration. Moreover, in α-syn PFFs-injected nNOS KO mice, no DA neuronal death was observed, suggesting that NO is a key mediator of α-syn PFFs-associated pathogenesis. 

In PD, NO plays an important role in a variety of signaling cascades that are crucial for maintaining the physiological functions of DA neurons [27]. Although it is not fully understood how NO contributes to neurodegeneration, NO and its derivatives are considered to be involved in the pathogenesis of PD. Indeed, numerous studies have shown that NO may cause glutamate-mediated excitotoxicity, DNA damage, and post-translational modification of proteins such as S-nitrosylation [28,29,30]. For example, inactivation of parkin, an E3 ubiquitin ligase, is associated with autosomal recessive juvenile PD as well as sporadic PD, and S-nitrosylation on multiple cysteine residues in RING and IBR domains of parkin disrupts its activity, leading to DA neuronal loss in sporadic PD [31,32,33]. In addition, dysfunction of the deubiquitinating protein Uch-L1 has been reported in PD [34]. S-nitrosylation of Uch-L1 at cysteine 152 inhibits its deubiquitinating activity by disturbing its interaction with ubiquitin. Moreover, S-nitrosylated Uch-L1 is structurally unstable, potentially serving as a seed and accelerating the aggregation of α-syn [35]. We observed that the transcriptional repressor PARIS can be regulated by S-nitrosylation. Since PARIS acts as a transcription factor for PGC-1α, we assessed the level of PGC-1α mRNA in PARIS overexpressing cells in the presence of nitrosative stress to find out whether S-nitrosylation on PARIS affects its transcriptional activity. Interestingly, we did not observe any reduction in PGC-1α mRNA due to SNO-PARIS; however, soluble PGC-1α protein was significantly decreased in the presence of SNO-PARIS, indicating that SNO-PARIS mediates the sequestration of functional PGC-1α into the insoluble deposits. 

PGC-1α is localized in both the cytoplasm and the nucleus, whereas oxidative stress triggers the nuclear accumulation of PGC-1α, and this subcellular localization of PGC-1α is regulated by SIRT1 [36]. In the Alzheimer’s disease model, soluble β-amyloid peptide oligomers (AβOs) prevent the PGC-1α-SIRT1 interaction, thereby decreasing the level of nuclear PGC-1α and promoting irreversible neurodegeneration [37]. In recent years, it has been shown that several human diseases are associated with mitochondrial dysfunction [38]. Since PGC-1α acts as a critical regulator of mitochondrial biogenesis [39], understanding the physiological role of PGC-1α is crucial for developing novel therapeutic interventions. Methylation of the PGC-1α promoter leads to a reduction in mitochondrial size and number along with the dysfunction of respiratory chain components [40]. In PD, dysregulation of PD-related proteins such as parkin, PINK1, and α-syn results in PGC-1α decrease and mitochondrial dysfunction in the SN region [41,42]. Indeed, downregulation of PGC-1α by PARIS inhibits mitochondrial biogenesis [43]. Moreover, PGC-1α is regulated by PINK1 via parkin-PARIS-PGC-1α, and it is an important mechanism for dopaminergic neuronal survival [44]. S-nitrosylation plays a crucial role in regulation of transcription factors, including NF-κB, HIF-1, and AP-1 [45,46,47]. S-nitrosylation of the transcription factor MEF2C during nitrosative/oxidative stress conditions in α-syn A53T-overexpressing DA neurons inhibits the MEF2C-PGC1α transcriptional mechanism, contributing to mitochondrial dysfunction and apoptotic cell death [48].

## 5. Conclusions

**Figure 8:**
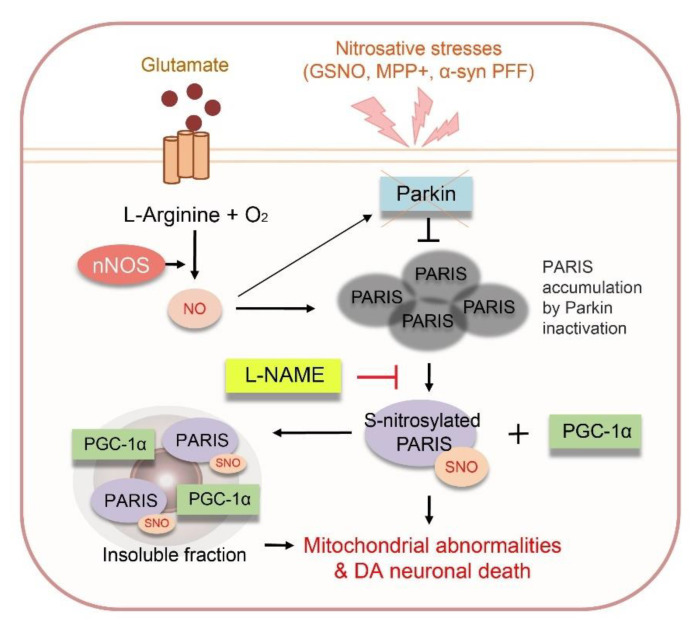
A proposed model of the function of the S-nitrosylated PARIS in dopaminergic neurons. PARIS can be S-nitrosylated at cysteine 265 (C265), and S-nitrosylated PARIS translocates to the insoluble fraction, leading to the sequestration of PGC-1α into insoluble deposits. In the sporadic PD model, α-synuclein preformed fibrils (α-syn PFFs)-injected mice, we found an increase in S-nitrosylated PARIS and insoluble sequestration of PGC-1α in substantia nigra (SN), resulting in mitochondrial abnormailities and DA neuronal death that were restored by N(ω)-nitro-L-arginine methyl ester (L-NAME). These results suggest that modulation of NO can be a therapeutic for α-syn PFFs-mediated neurodegeneration.

In summary, we demonstrated that PARIS can be S-nitrosylated at cysteine 265 residue, and S-nitrosylated PARIS (SNO-PARIS) translocates to the insoluble fraction, resulting in the sequestration of PGC-1α into the insoluble deposits (Figure 8). In α-syn PFFs-injected mice model, PARIS, SNO-PARIS, and insoluble sequestration of PGC-1α were increased, leading to the reduction of mitochondrial DNA copy number and ATP concentration that were blocked by L-NAME, a nitric oxide synthase (NOS) inhibitor. Similarly, α-syn PFFs-mediated increment of PARIS, SNO-PARIS, DA toxicity, and behavioral deficits were completely prevented in neuronal NOS KO mice, suggesting that α-syn PFFs-mediated nitrosative stress plays a crucial role in neurodegeneration. 

## Data Availability

Not applicable.

## Supplementary Materials

The following supporting information can be downloaded at: https://www.mdpi.com/article/10.3390/cells11223682/s1, Table S1: Antibodies used in this study; Table S2: Primers used in this study.
