# Supplementary material for: S-nitrosylated PARIS Leads to the Sequestration of PGC-1α into Insoluble Deposits in Parkinson’s Disease Model
Source: Cells. 2022 Nov 19;11(22):3682. doi: 10.3390/cells11223682 (PMC9688248; doi:10.3390/cells11223682)
Supplement: Supplementary file 1 [file cells-11-03682-s001.zip › cells-1940491-supplementary.pdf]

S-nitrosylated PARIS leads to the sequestration of PGC-1α into insoluble deposits in Parkinson's disease model

Hanna Kim, Ji-Yeong Lee, Soo Jeong Park, Eunsang Kwag, Jihye Kim, and Joo-Ho Shin

*Joo-Ho Shin
Email: jshin24@skku.edu

This PDF file includes:

Tables S1 to S2

Table S1. Antibodies used in this study.

Reagent	Source	Identifier
Rabbit polyclonal anti-ZNF746 (1:5000)	Proteintech	CAT#24543-1-AP
HRP-Streptavidin Conjugate (1:3000)	Invitrogen	CAT#43-4323
Mouse monoclonal anti-beta Actin [AC-15](HRP) (1:10000)	Abcam	CAT#ab49900
Anti-S-nitrosocysteine antibody [HY8E12] (1:1000)	Abcam	CAT#ab94930
Mouse monoclonal anti-PGC-1α (1:1000)	Calbiochem	CAT#ST1202
Rabbit polyclonal anti-Tyrosine Hydroxylase (1:1000)	Novus Biologicals	CAT#NB300-109
Mouse monoclonal anti-α-Synuclein Phospho (Ser129) (1:1000)	Biolegend	CAT#825702
Rabbit polyclonal anti-NRF1 (1:1000)	Abcam	CAT#ab34682
Mouse monoclonal anti-Parkin (Prk8) (1:1000)	Cell Signaling Technology	CAT#4211
Mouse monoclonal anti-GFP(B-2) (1:3000)	SANTA CRUZ BIOTECHNOLOGY	CAT#sc-9996
Mouse monoclonal ANTI-FLAG M2 (1:5000)	Sigma-Aldrich	CAT#F1804
donkey anti-goat IgG-HRP (1:5000)	SANTA CRUZ BIOTECHNOLOGY	CAT#sc-2020
c-Abl Antibody (1:3000)	Cell Signaling Technology	CAT#2862
Phospho-c-Abl (Tyr245) Antibody (1:3000)	Cell Signaling Technology	CAT#2861
Donkey Anti-Rabbit IgG H&L (Alexa Fluor® 488) (1:500)	Abcam	CAT#ab150073
Donkey Anti-Mouse IgG H&L (Alexa Fluor® 594) (1:500)	Abcam	CAT#ab150108

Table S2. Primers used in this study.

Target genes	Use		Human Primers (5’ – 3’)
β-actin	mt DNA	F	5’-CATGTGCAAGGCCGGCTTCG-3’
		R	5’-CTGGGTCATCTTCTCGCGGT-3’
ND1	mt DNA	F	5’-TCTCACCATCGCTCTTCTAC-3’
		R	5’-TTGGTCTCTGCTAGTGTGGA-3’
COX	mt DNA	F	5’-TTCGCCGACCGTTGACTATTCTCT-3’
		R	5’-AAGATTATTACAAATGCATGGGC-3’
PARIS (C265S)	SD	F	5’-CAT CCC TCT TCA GCA AGC TCG GAC GGG A-3’
		R	5’-TCC CGT CCG AGC TTG CTG AAG AGG GAT G-3’
PARIS (C265W)	SD	F	5’-CCT CTT CAG CAT GGT CGG ACG GGA CCC-3’
		R	5’-GGG TCC CGT CCG ACC ATG CTG AAG AGG-3’
Fragment 1	PARIS truncation	F	5’-GAATTCatggccgaggcggtcgcg-3’
		R	5’-GTCGACtcagatccagaagttcctgtt-3’
Fragment 2	PARIS truncation	F	5’-GAATTCctgcggctgcccccgggc-3’
		R	5’-GTCGACtcaaacatctggaatcttggg-3’
Fragment 3	PARIS truncation	F	5’-GAATTCcctgtggaccccagtcca-3’
		R	5’-GTCGACtcaactagggaagaaccgtgt-3’
Fragment 4	PARIS truncation	F	5’-GAATTCcctgcccaggaaggagcc-3’
		R	5’-GTCGACtcatggccttggggcctggct-3’
Fragment 5	PARIS truncation	F	5’-GAATTCttcaacgaaccctgtaaa-3G
		R	5’-GTCGACtcagctgccatcccgtgcgct-3G
Fragment 6	PARIS truncation	F	5’-GAATTCgcccttcggtgtggggag-3A
		R	5’-GTCGACtcacatgtccccgccatc-3T
PPARGC1A	RTQ	F	5’-TCCTCACAGAGACACTAGACA-3C
		R	5’-CTGGTGCCAGTAAGAGCTTCT-3T
**Target genes**	**Use**		**Mouse Primers (5’ – 3’)**
GAPDH	mt DNA	F	5’-TGGGTGGAGTGTCCTTTATCC-3’
		R	5’-TATGCCCGAGGACAATAAGG-3’

COX	mt DNA	F	5’-GCCTTTCAGGAATACCACGA-3’
		R	5’-AGGTTGGTTCCTCGAATGTG-3’
CYTB	mt DNA	F	5’-ATTCCTCATGTCGGACGAG-3’
		R	5’-ACTGAGAAGGCCCCCTCAAAT-3’
PPARGC1A	RTQ	F	5’-AGCCGTGACCACTGACAACGAG-3’
		R	5’-GCTGCATGGTTCTGAGTGCTAAG-3’
NRF1	RTQ	F	5’-GTTGGTACAGGGGCAACAGT-3’
		R	5’-TCGTCTGGATGGTCATTTCA-3’
TFAM	RTQ	F	5’-CCAAAAAGACCTCGTTCAGC-3’
		R	5’-CTTCAGCCATCTGCTCTTCC-3’
